# Intramuscular Olanzapine in the Management of Behavioral and Psychological Symptoms in Hospitalized Older Adults: A Retrospective Descriptive Study

**DOI:** 10.1155/2015/570410

**Published:** 2015-05-24

**Authors:** Silvia Duong, Kam-Tong Yeung, Feng Chang

**Affiliations:** ^1^North York General Hospital, 4001 Leslie Street, Toronto, ON, Canada M2K 1E1; ^2^Herzl CRIU Walk-In Centre, Jewish General Hospital, 5858 Côte-des-Neiges, Suite 500, Montreal, QC, Canada H3S 1Z1; ^3^School of Pharmacy, University of Waterloo, 200 University Avenue West, Waterloo, ON, Canada N2L 3G1

## Abstract

*Background*. While behavioral and psychological symptoms are frequent in hospitalized older adults with dementia or delirium, data supporting the off-label use of intramuscular atypical antipsychotics remain scarce. We examined the use of short-acting intramuscular (IM) olanzapine in hospitalized older adults to manage behavioral and psychological symptoms. *Methods*. A retrospective observational study of inpatients 65 years or older with at least one order for olanzapine IM during admission in urban Ontario Canada was conducted. Patient demographics, prescriptions for olanzapine IM, reason for administration, perceived effectiveness, adverse events, concurrently prescribed psychotropics, comorbidities, and patient discharge destination were recorded. *Results*. Among 82 patients aged 65–96 years (mean ± SD 79.3 ± 7.7) 85 cases were identified. Cognitive impairment or dementia affected 63.5% and 50.6% had comorbidities. Olanzapine IM was ordered 102 times and 34 patients (41%) received at least one dose. The intended efficacy was achieved in 79.4% of 78 cases of 124 doses given (62.9%). Fourteen (41%) patients who received doses experienced adverse events, with sedation and hypotension being the most common. *Conclusions*. Olanzapine IM appears effective in hospitalized older adults but is associated with potential adverse events. Structured monitoring and documentation are needed to ensure safe use in this high-risk population.

## 1. Introduction

Behavioral and psychological symptoms, such as agitation, aggression, wandering, delusions, and hallucinations, are often observed in older patients with dementia or delirium. Dementia is estimated to affect 7% of Canadians over the age of 60 and 55% of these patients are over the age of 80 [1]. Since the proportion of the population that is over the age of 80 is projected to continue to rise over the next 30 years, the number of people affected by dementia is expected to increase [1, 2]. Ninety percent of afflicted people experience neuropsychiatric or behavioral symptoms during the course of the disease [3]. Furthermore, dementia is associated with increased risk of developing delirium in hospitalized older patients: 10–15% of hospitalized seniors are admitted to hospital with delirium and 15–25% will develop delirium following admission [1]. Thus, the aging of the population also predicts that an increased number of elderly patients will exhibit behavioral and psychological symptoms.

The current evidence supporting drug treatment of behavioral and psychological symptoms in the elderly is scarce. Antipsychotics and benzodiazepines have been proposed as treatment options for the acute management of delirium [4]. Pharmacotherapy options for behavioral and psychological symptoms of dementia include antipsychotics, anticonvulsants, antidepressants, and anxiolytics. While oral therapy is the preferred route of administration in the management of behavioral and psychological symptoms due to its convenience, intramuscular formulations are often required when a patient refuses to take oral medications, is unable to swallow, or is too agitated to comply. The fast onset of parenteral administration also reduces the risk of potential harm to both the patient and others. As a result, clinicians commonly use intramuscular formulations of first-generation antipsychotics, benzodiazepines, or a combination of both for this purpose especially when no intravenous access is available. However, the potential for adverse events remains of concern especially in the more vulnerable and susceptible elderly population. Sedation, confusion, and respiratory depression are associated with benzodiazepines. First-generation antipsychotics may cause acute dystonia, extrapyramidal symptoms (EPS), neuroleptic malignant syndrome, and electrocardiographic abnormalities including QTc interval prolongation. Since they are associated with a lower risk of EPS, atypical or second-generation antipsychotics have become attractive alternatives over first-generation antipsychotics: a recent survey study reports that 86.3% of US nursing home residents receive atypical antipsychotics for off-label indications [5].

Short-acting olanzapine IM (Zyprexa Intramuscular) is currently approved for the treatment of acute agitation associated with schizophrenia and bipolar mania in many countries including Canada and the United States [6]. In adults, olanzapine IM has been shown to be efficacious in the management of agitation associated with schizophrenia, borderline personality disorder, bipolar disorder, and drug- and alcohol-using patients [7–12]. However, the use of olanzapine IM for the acute management of behavioral and psychological symptoms observed in older adults with dementia or delirium remains off-label to date. One published randomized controlled trial (RCT) reports similar efficacy between olanzapine IM and lorazepam IM in reducing agitation in patients with dementia [13]. No study has yet compared olanzapine IM with the combination of haloperidol and lorazepam. With the scarcity of data directing care, research is needed to better understand how olanzapine IM is being used in the management of acute behavioral and psychological symptoms in this patient population. This study has three main objectives: (1) to describe the patient characteristics of hospitalized older adults who receive a prescription for olanzapine IM, (2) to identify prescribing patterns of olanzapine IM in this population, and (3) to report documentation practices related to efficacy and adverse drug events associated with olanzapine IM use.

## 2. Methods

A retrospective descriptive study was carried out at a general tertiary care hospital in Toronto, Canada. The hospital had approximately 300 beds including an acute psychiatric inpatient unit, acute geriatric medicine, intensive care (ICU), internal medicine and surgical units, and emergency. Using the Inpatient Pharmacy Services database (PharmNet), the investigators identified all patients prescribed olanzapine IM between September 1, 2008, and March 1, 2010. Patients were included if they were at least 65 years of age and had at least one order for olanzapine IM during their hospital stay. The medical records of all cases were retrospectively reviewed and analyzed.

While all cases that were prescribed olanzapine are included in this review, we analyzed only those who actually received a minimum of one dose of olanzapine. The records of the remaining patients who were prescribed olanzapine but did not actually receive any are included separately for completeness.

Data collected included demographic characteristics, the prescribing patterns of olanzapine IM (indication, dose, frequency, and duration of therapy), relevant concurrently prescribed psychotropic medications (other prescribed antipsychotics, benzodiazepines, antidepressants, antidementia medications, mood stabilizers, pain medications, and other sedative-hypnotics) ordered within 24 hours of the initial prescription for olanzapine IM, comorbidities, and patient discharge destination. All comorbidities that could potentially contribute to patients' behavioral and psychological symptoms were recorded. These included cognitive impairment or dementia, delirium, psychiatric disorders (schizophrenia, bipolar affective disorder, and depression), neurological disorder (stroke and Parkinson's disease), insomnia, pain, and acute infection. Any unlisted potential factors were included if deemed applicable by consensus among investigators. The documented reason of administration and any findings on the effectiveness after dose were also noted. Subjective assessments based on clinician documentation were categorized as “achieved effect,” “partial effect” (adjunct therapy or repeated dose was required), “no effect,” or “not documented.” Documentation for adverse events associated with olanzapine IM including hypotension, falls, decreased level of consciousness, EPS, weakness, sedation, and potential events such as infections was reviewed. All monitoring for vital signs (temperature, heart rate, blood pressure, respiration rate, and oxygen saturation) within six hours after dose was also included. The duration of therapy was determined by counting the number of days with an active order for olanzapine IM according to Physician's Order Sheet and the Medication Administration Record.

A standardized data abstraction form in Microsoft Excel was used and pilot-tested. Data were managed with Excel for descriptive analysis. This study was conducted with approval from the North York General Hospital Research Ethics Board.

## 3. Results

A total of 87 cases met the inclusion criteria; however, two charts were neither available nor accessible at the time of the data collection. Eighty-five cases were found among 82 older inpatients (two patients were readmitted once (*n* = 1) or twice (*n* = 1) during the course of the study and ordered olanzapine IM during each admission). Most cases (52; 61.2%) were admitted to the psychiatric ward while 31 (36.4%) were from internal medicine. Other cases came from surgical, transitional, and intensive care units (2.4%). Of the 85 cases, 51 (60%) had an order for olanzapine IM but did not require any doses, while 34 (40%) received at least one dose. There were slightly more women (52.9%) than men (47.1%). The age ranged from 65 to 96 years (mean ± SD age, 79.3 ± 7.7 years). Two patients who were prescribed olanzapine, but who did not receive any, passed away during admission due to sepsis and sepsis-related complications. Detailed demographic data and clinical characteristics of both those who did and did not receive olanzapine are presented in Table 1. Most suffered from cognitive impairment or dementia (63.5%) and about half (50.6%) had two or more comorbidities. In terms of concurrent pharmacotherapy, most cases were prescribed another antipsychotic (83.4%) and other psychotropic medications (75.3%), with olanzapine oral (41%), risperidone (40%), and quetiapine (22%) most commonly prescribed.

During the study period, 102 orders for olanzapine IM were written for these 85 cases, mostly initiated by a psychiatrist (82.4%) for the following two indications: agitation as assessed and documented by a clinician and/or patient's refusal or inability to receive oral antipsychotics (Table 2). The prescribed dose of olanzapine IM ranged from 2.5 mg to 10 mg per injection (mean ± SD, 5.51 ± 1.91 mg). All prescriptions were written on an “if needed” (PRN) basis with the frequency of administration varying from every 1 hour to every 12 hours. Three (2.9%) orders had a frequency of every 1 hour. A maximum daily dose (5–20 mg/day) was prespecified in 25.5% of the orders only.

Among the 34 cases who received at least one dose of olanzapine IM, the most common reasons for administering included patient's refusal or inability to take oral antipsychotics (33.9%), agitation and patient's refusal/inability to take oral medication (33.9%), and aggression and patient's refusal/inability to take oral medications (22.6%). Patients received 1 to 3 doses (dosage varying between 2.5 mg and 10 mg) per day and 1 to 24 doses throughout admission (3.3 ± 4.5 doses). The total dose administered during admission varied between 2.5 and 120 mg (12.1 ± 20.1 mg). In 46 (37.1%) of 124 administered doses, the effectiveness was not documented in the patients' chart. For the remaining 78 doses, 68 (87%) achieved the intended or partial effect. Five (6.4%) had no effect and five (6.4%) were undetermined. Doses of 2.5 mg and 5 mg were sufficient in achieving effectiveness in 82.1% and 80% of cases, respectively.

Fourteen of the 34 cases (41.2%) documented experiencing at least one adverse event associated with the administration of olanzapine IM. Sedation and hypotension were the most commonly reported (Table 3). Four patients (11.8%) experienced a fall within a period of 24 hours after dose and 11 patients suffered from an infection after receiving at least one dose (six cases of a urinary tract infection, three cases of pneumonia, and one case each of cellulitis and* Clostridium difficile*-associated infection). Monitoring for patient's vital signs within six hours after dose was completed in about one-third of cases.

## 4. Discussion

To our knowledge, this is the first study to report the clinical use of short-acting olanzapine IM for its off-label use in the management of behavioral and psychological symptoms in hospitalized older adults. In our center, olanzapine IM was commonly prescribed to manage acute agitation and aggression related to dementia, delirium, or psychiatric disorders. It was also used as an alternative when patients refused or were unable to take oral antipsychotics. In a double-blind, placebo-controlled study of 272 patients (mean age 77) with Alzheimer's disease, vascular or mixed dementia, Meehan and colleagues demonstrated that 5 mg of olanzapine IM had similar efficacy to 1 mg of lorazepam IM in reducing agitation [13]. In contrast to this previous study, 50% of the older inpatients in our present study suffered from two or more comorbidities that can cause acute behavioral and/or psychological symptoms. Furthermore, most patients received concurrent oral antipsychotics or other psychotropic medications.

Currently, olanzapine is the only intramuscular short-acting second-generation antipsychotic formulation available on the Canadian market. In the USA, intramuscular formulations for ziprasidone and aripiprazole are short-acting alternatives for the management of acute agitation. In the older population, ziprasidone has been reported to be effective in short-term treatment of acute psychosis or agitation, offering similar effectiveness as conventional therapy (haloperidol with or without lorazepam) [14, 15]. A recent double-blind randomized placebo-controlled study in a long-term care setting demonstrated that aripiprazole is effective in the management of agitation associated with Alzheimer's, vascular or mixed dementia [16]. Furthermore, in a randomized controlled trial, Baldaçara and colleagues compared the effectiveness of intramuscular olanzapine, ziprasidone, haloperidol plus promethazine, haloperidol plus midazolam, and haloperidol alone for the treatment of patients (mean age 32.2 years) with agitation and aggressive behavior in the emergency rooms for psychiatric patients [17]. The authors concluded that all were effective in controlling agitation/aggressive behaviors associated with psychotic or bipolar disorder over a period of 12 hours. Among the different study groups, they noted that patients receiving olanzapine had better agitation control, experienced less excessive sedation, and were less likely to require physical restraint. Since the efficacy and safety of two second-generation intramuscular antipsychotics in management of acute agitation in the elderly have never been directly compared, it is impossible to conclude whether a particular agent is preferred in the geriatric population.

In our study, the 2.5 mg and 5 mg doses of olanzapine IM appeared to achieve efficacy in about 80% of the documented cases. Breier and colleagues reported that 2.5 mg of olanzapine IM was less efficacious than haloperidol IM 7.5 mg and higher doses of olanzapine IM (5 mg, 7.5 mg, and 10 mg) in the management of acute agitation in adult patients (mean age 36.3 years, range from 18 to 73 years) with schizophrenia [7]. Response rates ranged from 50% to 80% with olanzapine 2.5 mg to 10 mg. Conversely, Meehan and colleagues reported that olanzapine IM 2.5 mg and 5 mg were superior to placebo and offered similar efficacy as lorazepam IM 1 mg in alleviating acute agitation in patients with dementia [11]. An initial treatment dose of 2.5 mg/day has also been previously proposed in older patients with Alzheimer's disease [13]. Our data suggested that olanzapine IM 2.5 mg offered similar efficacy as 5 mg in these older patients. Prescribers may consider initiating therapy at the lower dose of 2.5 mg and provide a second dose to be given at least two hours after if needed [13]. The Zyprexa IM product monograph recommends a maximum daily dose of 20 mg [6]. Since renal and hepatic impairments are common in the elderly, a more conservative maximum daily dose of 10 mg may be considered for hospitalized elderly patients.

In terms of adverse events, previous studies reported high prevalence of sedation and orthostatic hypotension with the use of olanzapine IM [7, 13]. Importantly, older adults also experience decreased level of consciousness and increased risk of falls. Close monitoring of vitals, level of consciousness, and gait stability are recommended to detect and, hopefully, prevent adverse events. Marder and colleagues identified that the most common postmarketing adverse events associated with olanzapine IM included pyrexia, hypotension, syncope, neuroleptic malignant syndrome, somnolence, cardiac arrest, rise in the serum creatine phosphokinase (CPK), and falls [18]. Adverse events were frequently reported in patients with comorbid conditions and those receiving concomitant treatment with benzodiazepine (39%) and other antipsychotic agents (54%) [18]. Therefore, in the susceptible older population, clinicians should attempt to reduce the risk of potential adverse events by exercising caution when prescribing olanzapine IM concomitantly with other psychotropic medications. In our study, a significant portion of patients did not have documentation relevant to efficacy or safety. The development of an appropriately structured postdose monitoring protocol that includes neurological assessment, mobility assessment, fall prevention strategies, and swallowing assessment would ensure safer use. Vital signs should be taken within six hours after a given dose if clinically feasible.

As with any retrospective study, our data are limited by the availability and accuracy of medical records. The lack of documentation in 37.1% of administered doses of olanzapine reduces the ability to draw firm conclusions about the safety and effectiveness of its use in this vulnerable population. A comparator group would have permitted us to more accurately assess the efficacy and risk of adverse events associated with olanzapine IM. Also, our data are reflective of one site only and practice may differ in other settings. However, this study does provide a glimpse into the clinical use of olanzapine IM among hospitalized older adults in naturalistic acute care practice.

Results can be used to direct future prospective studies comparing management strategies. In these future studies, it is recommended that agitation be measured objectively rather than subjectively, so that multisite comparisons can be made. Examples of agitation measurement tools used with people with dementia include the Agitated Behavior Scale (ABS), the Pittsburgh Agitation Scale, and the Cohen-Mansfield Agitation Inventory. Consistent use of such a tool will enable a thorough assessment of benefit versus risk. Future studies focusing on surgical and ICU patient populations can further understanding for the dosing approach required by medically compromised patients.

## 5. Conclusions

Olanzapine IM appears to be commonly prescribed for behavioral and psychological symptoms in older patients in the acute care setting. The use of olanzapine IM appears effective; however, its administration is associated with a significant risk of potentially related adverse events. A structured approach to monitoring and documentation will be needed to ensure safe use.

## Figures and Tables

**Table 1:** Baseline demographic characteristics of older patients prescribed olanzapine.

	Received olanzapine	No olanzapine	All cases
	*N* = 34	*N* = 51	*N* = 85
Age, mean (years)	79.6	79.0	79.3
Female	19 (55.9%)	26 (51%)	45 (52.9%)
Admission from			
Home	18 (52.9%)	41 (80.4%)	59 (69.4%)
Another hospital	4 (11.8%)	2 (3.9%)	6 (7.1%)
Retirement/LTC facilities	12 (35.3%)	8 (15.7%)	20 (23.5%)
Discharge destination			
Home	10 (29.4%)	20 (39.2%)	30 (35.3%)
Another institution	6 (17.6%)	6 (11.8%)	12 (14.1%)
Retirement/LTC facilities	18 (53.0%)	23 (45.1%)	41 (48.2%)
Death	0	2 (3.9%)	2 (2.4%)
Types of patients			
Psychiatry	22 (64.7%)	29 (56.9%)	51 (60.0%)
General medicine	12 (35.3%)	21 (41.2%)	31 (36.4%)
Surgery	0	2 (3.9%)	2 (2.4%)
Intensive care unit	0	1 (2.0%)	1 (1.2%)
Comorbidities			
Cognitive impairment/dementia	21 (61.8%)	33 (64.7%)	54 (63.5%)
Delirium	9 (26.5%)	16 (31.4%)	25 (29.4%)
Psychiatric disorders	19 (55.9%)	24 (47.1%)	43 (50.6%)
Neurological disorder	5 (14.7%)	6 (11.8%)	11 (12.9%)
Insomnia	3 (8.8%)	5 (9.8%)	8 (9.4%)
Pain	0	1 (1.9%)	1 (1.2%)
Acute infection	7 (20.6%)	13 (25.5%)	20 (23.5%)
Other	6 (17.6%)	5 (9.8%)	11 (12.9%)
Number of comorbidities			
1	17 (50.0%)	25 (49.0%)	42 (49.4%)
2	11 (32.3%)	10 (19.6%)	21 (24.7%)
3	2 (5.9%)	10 (19.6%)	12 (14.1%)
≥4	4 (11.8%)	6 (11.8%)	10 (11.8%)
Concurrent psychotropic medications			
Other antipsychotics	29 (85.3%)	41 (80.4%)	70 (83.4%)
Benzodiazepines	11 (32.4%)	10 (19.6%)	21 (24.7%)
Antidepressants	18 (52.9%)	18 (35.3%)	36 (42.4%)
Antidementia medications	7 (20.6%)	14 (27.4%)	21 (24.7%)
Mood stabilizer	4 (11.8%)	4 (7.8%)	8 (9.4%)
Pain medications	0	1 (1.96%)	1 (1.2%)
Other sedatives	0	2 (3.9%)	2 (2.4%)
Number of psychotropic medications (nonantipsychotics)			
0	8 (23.5%)	13 (25.5%)	21 (24.7%)
1	9 (26.5%)	20 (39.2%)	29 (34.1%)
2	14 (41.2%)	17 (33.3%)	31 (36.4%)
3	1 (2.9%)	1 (2.0%)	2 (2.4%)
4	2 (5.9%)	0	2 (2.4%)

^*∗*^LTC: long-term care.

**Table 2:** Prescribing patterns of olanzapine IM.

	Received olanzapine	Received no olanzapine	All prescriptions
	*N* = 46	*N* = 56	*N* = 102
Indication(s) of therapy	Number (%)		
Refusal/unable to take oral meds	9 (19.6%)	7 (12.5%)	16 (15.7%)
Agitation	3 (6.5%)	3 (5.3%)	6 (5.9%)
Others	2 (4.3%)	1 (1.8%)	3 (2.9%)
Combination of indications			
Agitation and/or refusal/unable	12 (26.1%)	22 (39.3%)	34 (33.3%)
Agitation, refusal/unable, and/or aggression	9 (19.6%)	12 (21.4%)	21 (20.6%)
Agitation and/or aggression	4 (8.7%)	8 (14.3%)	12 (11.8%)
Psychosis and/or agitation	1 (2.2%)	2 (3.6%)	3 (2.9%)
Psychosis, agitation, and/or aggression	3 (6.5%)	0 (0.0%)	3 (2.9%)
Psychosis, agitation, and/or refusal/unable	2 (4.3%)	0 (0.0%)	2 (2.0%)
Agitation and/or others	1 (2.2%)	0 (0.0%)	1 (1.0%)
Aggression and/or refusal/unable	0 (0.0%)	1 (1.8%)	1 (1.0%)
Duration of therapy (days)			
Mean ± SD	26.7 ± 39.6	14.1 ± 13.0	19.2 ± 27.80
Range	5–250	1–60	1–250

**Table 3:** Documented adverse events and monitoring among cases that received olanzapine IM.

Adverse event	Number (%)
Sedation	7 (20.6%)
Orthostatic hypotension	5 (14.7%)
Falls	4 (11.8%)
Decreased level of consciousness	4 (11.8%)
Extrapyramidal symptoms	3 (8.8%)
Weakness	1 (2.9%)
Monitoring of vital signs (within 6 hrs)	
Complete monitoring	42 (33.9%)
Incomplete monitoring	10 (8.1%)
No monitoring documented	58 (46.8%)
Patient refuses to comply	14 (11.2%)
